# Role of Epigenetic Histone Modifications in Diabetic Kidney Disease Involving Renal Fibrosis

**DOI:** 10.1155/2017/7242384

**Published:** 2017-06-13

**Authors:** Jing Sun, Yangwei Wang, Wenpeng Cui, Yan Lou, Guangdong Sun, Dongmei Zhang, Lining Miao

**Affiliations:** Department of Nephrology, Second Hospital of Jilin University, Changchun 130041, China

## Abstract

One of the commonest causes of end-stage renal disease is diabetic kidney disease (DKD). Renal fibrosis, characterized by the accumulation of extracellular matrix (ECM) proteins in glomerular basement membranes and the tubulointerstitium, is the final manifestation of DKD. The TGF-*β* pathway triggers epithelial-to-mesenchymal transition (EMT), which plays a key role in the accumulation of ECM proteins in DKD. DCCT/EDIC studies have shown that DKD often persists and progresses despite glycemic control in diabetes once DKD sets in due to prior exposure to hyperglycemia called “metabolic memory.” These imply that epigenetic factors modulate kidney gene expression. There is evidence to suggest that in diabetes and hyperglycemia, epigenetic histone modifications have a significant effect in modulating renal fibrotic and ECM gene expression induced by TGF-*β*1, as well as its downstream profibrotic genes. Histone modifications are also implicated in renal fibrosis through its ability to regulate the EMT process triggered by TGF-*β* signaling. In view of this, efforts are being made to develop HAT, HDAC, and HMT inhibitors to delay, stop, or even reverse DKD. In this review, we outline the latest advances that are being made to regulate histone modifications involved in DKD.

## 1. Introduction

One of the main causes of end-stage renal disease is diabetic kidney disease (DKD) [1]. Approximately 20% to 40% of diabetic patients eventually develop DKD. Although the exact cause of DKD is unknown, several factors including genetic, environmental, and hemodynamic factors; high blood glucose; high blood lipids; hypertension; and proteinuria contribute to its development [2]. All these factors appear to modulate the production and action of various growth factors/cytokines and reactive oxygen species (ROS), which will give rise to podocyte damage and interstitial inflammation that participate in the pathogenesis of DKD. This complex set of events ultimately leads to glomerular dysfunction and renal failure due to the deposition of excess extracellular matrix (ECM) proteins and increase in renal glomerulosclerosis [2, 3]. Chronic and relentless fibrosis in both glomerular and tubulointerstitial compartments are characterized by ECM accumulation and an increase in the deposition of collagen, fibronectin, and laminin in mesangial matrix, glomerular basement membranes, and tubulointerstitium, which are pathologic manifestations of DKD [4]. It is known that even after control of hyperglycemia, diabetic patients may continue to develop renal complication with glomerular and tubulointerstitial fibrosis, eventually leading to renal failure [5]. These evidences suggest that epigenetics may have a significant role in the pathobiology of DKD [6].

## 2. Role of Renal Fibrosis in Diabetic Kidney Disease

The accumulation of ECM proteins is the hallmark of DKD [4, 7]. This is supported by observation that collagens, fibronectin, and laminin are deposited in increased amounts in the glomerular basement membrane and mesangial extracellular matrix, even in the early stages of DKD (microalbuminuria stage), leading to the occurrence of diabetic diffuse glomerulosclerosis [4, 8–10]. In the late stages of diabetic glomerulosclerosis, which is called Kimmelstiel-Wilson lesion, the deposition of type I and III collagens increase severely [11, 12]. Type IV collagen in both serum and urine have been demonstrated to increase in the early and established stages of DKD [13–15]. Thus, it can be seen that accumulation of ECM proteins work throughout the whole process of the renal fibrosis in DKD.

TGF-*β*1, a broad-spectrum cytokine [16, 17], which could be induced by hyperglycemia, advanced glycation end-products, mitogen-activated protein kinase (MAPK), and protein kinase C (PKC) pathway [18], plays a crucial role in the progression of glomerular enlargement and the excess deposition of ECM in DKD [19, 20]. Hyperlycemia-induced increase in the deposition of matrix proteins that result in glomerulosclerosis is due to the increased expression and activation of TGF-*β*1 in glomerular cells, podocytes [21], and mesangial cells [22]. In addition, it can also stimulate *α*-smooth muscle actin (*α*-SMA), collagen type I expression, and cell hypertrophy [23, 24]. Connective tissue growth factor (CTGF) appears to be a downstream molecule of TGF-*β*1, ultimately leading to renal fibrosis after activation [25–27]. It has been shown that the profibrotic action of TGF-*β*1 could be blocked by CTGF antisense oligonucleotides [28]. It is investigated that after activation, CTGF can increase the expression of fibronectin and collagens IV, III, and I and facilitate the deposition and assembly of ECM proteins [29, 30].

It has been shown that activated myofibroblasts are the principal effector cells, and its number could be correlated with the excess deposition of interstitial ECM in DKD [31]. However, the precise origin and activation procedure of myofibroblasts in fibrotic kidneys remains unclear. Studies by Galichon and Hertig [32] revealed that myofibroblasts might arise from tubular epithelial cells through epithelial-to-mesenchymal transition (EMT) in the process of renal fibrosis. In general, it is believed that the transformation of impaired tubular epithelial cells into mesenchymal cells is the most probable mechanism associated with the development of fibrosis in DKD [31]. Both in vitro and in vivo studies revealed that EMT could be triggered by a number of agents, in which profibrotic element transforming growth factor-*β*1 (TGF-*β*1) appears to be a major player [33, 34].

## 3. Diabetes Nephropathy and Metabolic Memory

It is known that despite achieving glycemic control, patients with diabetes, who experienced prior exposure to high glucose levels, continue to develop diabetic complications including DKD. This manifestation is known as “metabolic memory.” In the Diabetes Control and Complications Trial (DCCT), wherein conventional versus intensive insulin therapy were compared in type I diabetic patients, it was observed that intensive insulin regimen reduced the incidence or severity of diabetic renopathy, peripheral neuropathy, and retinopathy compared to patients who were on nonintensive insulin therapy [35]. When the DCCT cohort was examined annually for the next eight years, as part of the follow-up to understand the long-term consequences of therapies instituted, referred to as Epidemiology of Diabetes Interventions and Complications (EDIC) study, it was noted that patients who maintained strict glycemic control during the DCCT had a sustained benefit in postponing the advancement of DKD. It was also observed that DKD progression was much less aggressive in the strict glycemic control group compared to the conventional group, even though HbA1c levels did not substantially differ between these two groups during the EDIC phase [36]. In support of these results, it was reported that type 2 diabetic patients, who were under intensive glycemic control, exhibited long-term benefits in the form of a decrease in both macro­ and microvascular complications, which was referred to as the “legacy effect” [37].

Furthermore, studies performed in experimental animal models also provided additional support to this concept of “metabolic memory.” For instance, Li et al. [38] manifested that vascular smooth muscle cells obtained from the aortas of db/db mice (type 2 diabetes) exhibited upregulated proinflammatory responses compared to controls (db/+ mice that were nondiabetic). Similarly, type 1 diabetic rats that had hyperglycemia for several weeks prior to inducing normoglycemia revealed the progression of DKD [39]. Homologous evidence with diabetic rats confirmed that despite islet transplantation (that lasted for at least 12 weeks), which had their hyperglycemia reverted to normoglycemia after six weeks of diabetes, progression of diabetic retinopathy is continuously shown [40]. Previous observations confirm that “metabolic memory” plays a key role in the development of diabetic complications, which calls for more in depth and intense studies to determine the molecular mechanisms underlying this process. These evidences led to the belief that epigenetic factors may have an effect in the pathobiology of “metabolic memory” and diabetic complications.

## 4. Epigenetics and Histone Modifications

Waddington [41] originally defined epigenetics as being responsible for programmed changes during embryonic development, as a result of “the causal interaction(s) between genes and their products that brings in a change in phenotype.” The epigenome acts as a bridge between genetics and the environment, and the epigenetic code modifies gene expression to determine the final phenotype without alterations in DNA sequences [42]. Epigenetic modifications could change disease phenotype by affecting the target gene directly, as a reply to environmental signals and pathologic states such as diet, exercise, toxins, oxidative stress, inflammation, and metabolic changes [43]. Epigenetic alterations of gene(s) have an important effect in the formation and development of embryo, X-chromosome inactivation, genomic imprinting, cell differentiation and identity, stable inheritance of gene expression, function of immune cells, plasticity of stem cells, and cellular responses to environmental signals [44, 45]. Histone modifications and DNA methylation, along with noncoding RNAs, are collectively known as epigenetic modifications that contain the epigenetic information needed for the stable heredity of gene expression prototypes in differentiated cells [45]. Recent investigations have revealed that epigenetic mechanisms play a significant role in the pathobiology of diabetes mellitus and DKD.

Histone modifications, in essence termed posttranslational modifications of histone in chromatin, are an important part of the epigenetic layer that maintains normal cellular transcriptional patterns [46]. Histone modifications mostly occur in the exposed histone amino-terminal tails such as lysine acetylation (Kac), lysine methylation (Kme), and ubiquitination, as well as threonine and serine phosphorylation and arginine methylation [47]. These produce modifications in histone tails and chromatin structure changes that lead to changes in the binding of transcription agents to their respective core promoter elements, resulting in the activation or suppression of special genes [48]. Recently, it was reported that histone modifications, especially histone acetylation and histone methylation modifications, seem to be important in the pathobiology of DKD, which would be discussed in this review.

**Figure 1:**
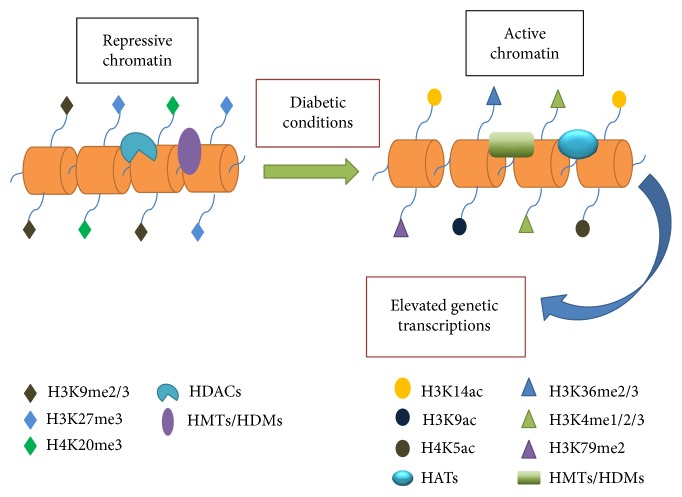
Histone modifications stimulate the gene expressions in diabetic conditions. In normal conditions, histone deacetylases (HDACs) and histone methyltransferases (HMTs)/histone demethylases (HDMs) recruit at the gene promoters, leading to the removal of acetylation and the accumulation of repressive chromatin markers (such as H3K9me2/3, H3K27me3, and H4K20me3) at the gene promoters and inhibiting the initiation of genetic transcriptions. While in diabetic conditions, the repressive histone modifications are cleared away and are replaced by the enrichment of active chromatin marks (histone acetylations and H3K36me2/3, H3K4me1/2/3, and H3K79me2), resulting in the upregulation of the expression of inflammatory and fibrotic genes and ultimately promoting the progress of diabetic renal complications. HDACs, histone deacetylases; HATs, histone acetyltransferase; HMTs, histone methyltransferase; HDMs, histone demethylases.

Histone acetylation, the acetylation of the N-terminal tails of H3K and H4K, is a reversible dynamic process [49]. In general, histone acetylation of the lysine site (such as H3K14ac, H3K9ac, and H4K5ac) of gene promoters stimulates transcriptional activation, while the removal of acetylation is relevant to gene repression [50] (Figure 1). Histone acetylation states are determined both by histone acetyltransferases (HATs) and histone deacetylases (HDACs). P300/CBP (CREB-binding protein), one of the predominant histone acetyltransferases catalyzed lysine acetylation, is a chromatin marker that results in gene activation. In another way, HDACs modulate the removal of acetylation and act as a repressor of gene transcription [45] (Figure 1). Furthermore, HDACs can be assorted into four classes, depending on their sequence similarity and cofactor interactions: class I (HDACs 1, 2, 3, and 8) is nuclear enzymes, extensively expressed in diverse tissue types; class II (HDACs 4, 5, 6,7, 9, and 10) and class IV (HDAC 11), which are chiefly located in the cytoplasm, are expressed in specific tissues; and class III embraces the sirtuin family (SIRT1–7) [51, 52]. The expression of the different family members of HDACs varies from tissue to tissue and exhibits different biological effects. In adult kidneys, all of class I and II HDAC members are expressed [52].

Histone methylation, in contrast to acetylation, is more constant and long-standing [48]. Histone methylation takes place on both arginine and lysine residues, which may be mono-, di-, or trimethylated. Histone methylation could be manifested as gene repression or activation depending upon the residue modified [53]. In general, H3K36me2/3, H3K4me1/2/3, and H3K79me2 are relevant to the activation of gene transcription. However, H3K9me2/3, H3K27me3, and H4K20me3 are considered as repressive chromatin markers [54] (Figure 1). Despite being relatively stable, histone methylation could be dynamically modified through the concerted action of histone methyltransferases (HMTs) and histone demethylases (HDMs) [55]. Due to varying specificities of numerous HDMs, their potential role in various diseases is currently being evaluated.

## 5. Histone Modifications Participate in the Progression of Diabetic Kidney Disease

Accumulating evidences show that epigenetic histone modification plays a significant role in modulating kidney gene expression under diabetic circumstances (Figure 1). In both in vitro and in vivo investigations related to diabetes, it has been demonstrated that histone lysine methylation and acetylation patterns changed, along with the recruitment of HATs/HDACs or HMTs at gene promoters [6, 48, 56, 57]. Histone hyperacetylation and increased H3K4me are implicated in the modification of islet-specific insulin gene expression in response to changing glucose levels, which correlated with p300 HAT and HMT SET7/9 recruitment [58, 59]. Knockdown of Jhdm2a gene, the H3K9me2 demethylase, has been reported to lead to obesity and hyperlipidemia, implying an important role of histone modifications in diabetes [60]. Monocytes from T1D and T2D patients have been found to have increased H3K9/14Ac in company with the recruitment of HAT p300/CBP at promoters of inflammatory genes TNF-*α* and COX-2, which resulted in the upregulation of the expression of these inflammatory genes [61]. It was reported that renal mesangial cells induced the transcription of fibrotic genes in reply to TGF-*β*1, and high glucose is due to the enrichment of active chromatin marks (H3K4me1/2/3, H3K9/14Ac) and the decrease of repressive markers (H3K9me2/me3) at promoters of these genes, along with the histone lysine acetyltransferase (p300/CBP) and histone lysine methyltransferase (SET7) occupancies at fibrotic gene promoters [62, 63]. On the other hand, these effects were significantly reversed by the HAT domain mutations of p300/CBP or SET7/9 gene silencing. Studies in animal models have also revealed epigenetic histone modifications in DKD. Global histone changes associated with the transcription of fibrotic genes related to DKD have also been described [44]. In type 1 diabetic mice, diabetes-induced increases in histone acetylation and HAT activity, as well as the enrichment of H3K9/14Ac and HAT p300/CBP at the fibrotic gene promoters contributed to the upregulation of the expression of fibrotic genes that were significantly and persistently attenuated by a novel curcumin analog C66 treatment [64]. These studies emphasize the critical role played by epigenetic histone modifications in the pathogenesis of diabetic kidney disease. A better apprehension of these variations in histone lysine acetylation and methylation may aid in the identification of new biomarkers and significant therapeutic targets for DKD.

## 6. Histone Modifications Involve Renal Fibrosis of DKD

A key element in DKD is the excess accumulation of ECM proteins comprising fibronectin, collagens, and laminin in the kidney [65]. It has been widely believed that EMT is triggered by TGF-*β*1, which is an outstanding mechanism in the progression of fibrosis due to DKD [66], and this can be countered and reversed by BMP-7 [34]. It has been demonstrated that the TGF-*β*1 has an important role in triggering EMT and the accumulation of ECM proteins in DKD [18, 67]. Histone modifications also influence the expression and regulation of pathways known to mediate renal fibrosis in DKD.

## 7. Histone Modifications Promote the Expression of Profibrotic Factors

**Figure 2:**
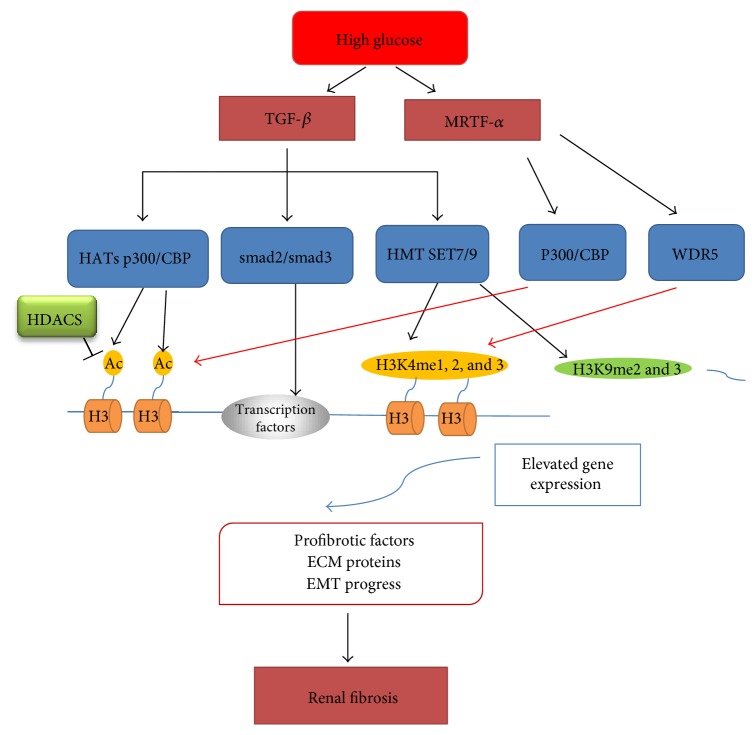
Histone modifications regulate gene transcription involving renal fibrosis of diabetic kidney disease. TGF-*β* signaling pathway, activated by high glucose, will stimulate two types of epigenetic histone mechanisms including histone acetylation and histone methylation. TGF-*β*1 activates the histone acetyltransferase (HAT) p300/CBP, followed by the enrichment of H3K9/14Ac and HAT p300/CBP at the promoters of renal fibrotic genes, and then increases the transcriptions of fibrotic genes and EMT progress. After induced by TGF-*β*1, the methyltransferase SET7/9 enriches at fibrotic gene promoters, leading to increases of positive chromatin marks, such as H3K4me1, H3K4me2, and H3K4me3, and reductions of inhibitive marks including H3K9me2 and H3K9me3, at promoters of fibrotic genes. Eventually, the expressions of renal fibrotic genes are upregulated, which will result in the progression of DKD. Another pathway, myocardin-related transcription factor A (MRTF-A), also can be activated by high glucose. MRTF-A can regulate the histone acetylation and histone methylation of renal fibrotic genes by accelerating the recruitment of HAT p300/CBP and HMT WDR5 to fibrotic gene promoters, resulting in transcriptional activation. DKD, diabetic kidney disease; TGF-*β*, transforming growth factor *β*; MRTF-A, myocardin-related transcription factor A; HAT, histone acetyltransferase; HDAC, histone deacetylase; HMT, histone methyltransferase; Smad, mothers against decapentaplegic homologue; ECM, excess extracellular matrix; EMT, epithelial-to-mesenchymal transition.

The profibrotic cytokines such as plasminogen activator inhibitor 1 (PAI-1), connective tissue growth factor (CTGF), and p21 play a significant role in the progression of excess deposition of ECM in DKD. A lot of studies demonstrated that the expressions of profibrotic cytokines were regulated by the histone modifications in diabetic condition (Figure 2). In cultured rat mesangial cell, high glucose and TGF-*β*1 induced elevation of active Kme marks (H3K4me1, 2, and 3) and decrease of inhibitive marks (H3K9me2 and 3) at PAI-1 and CTGF gene promoters, accompanied with the accumulation of HMT SET7/9 to fibrotic and ECM gene promoters, resulting in the increased expression of these profibrotic proteins [63]. Similarly, in TGF-*β*1 and high-glucose-treated rat mesangial cells, the enrichment of H3K9/14Ac and HAT p300/CBP at promoters of PAI-1 and p21 gene promoted the facilitation of PAI-1 and p21 production [62]. In a study using type 1 diabetic mice, renal CTGF and PAI-1 gene expressions were augmented by the activation of histone acetylation and HAT activity, as well as the enrichment of H3K9/14Ac and HAT p300/CBP at the promoters of CTGF and PAI-1 gene [64].

## 8. Histone Modifications Accelerate the Accumulation of ECM Proteins

ECM proteins such as collagen, laminin, and fibronectin, accumulated in mesangial matrix, glomerular basement membranes, and the tubulointerstitium, are pathologic features of DKD. Epigenetic histone modifications may involve in the progression of renal fibrosis of DKD by means of regulating the gene transcription of ECM proteins (Figure 2).

In both in vitro and in vivo diabetic kidney disease, collagen gene expressions can be regulated by histone modifications through the recruitment of HATs/HDACs and HMTs at collagen gene promoters, and histone lysine methylation and acetylation patterns changed. Several studies revealed that histone acetyltransferase p300 accelerates COL1A1/COL1A2 expression in numerous physiological and pathological cellular processes [57, 68–70]. Cultured tubular epithelial cells in diabetic conditions and kidneys of diabetic mice revealed an increased expression of myocardin-related transcription factor A (MRTF-A), which led to the recruitment of p300 and WDR5 to collagen promoters, resulting in transcriptional activation [71]. In contrast, MRTF-A silencing notably resulted in the disappearance of acetylated histone H3K18/K27 and trimethylated histone H3K4 indicative of transcriptional activation. Sun et al. [63] found that in mesangial cells of rat, TGF-*β*1 and high-glucose treatment increased the levels of positive chromatin marks, such as H3K4me1, H3K4me2, and H3K4me3, and reduced the levels of inhibitive marks including H3K9me2 and H3K9me3, at collagen-*α*1 gene promoters. These changes were found to be associated with the recruitment of H3K4 methyltransferase SET7/9 to collagen-*α*1 gene promoters, which eventually led to the upregulation of collagen-*α*1 gene expression. Through siRNA studies, it has been shown that SET7/9 gene silencing attenuates collagen-*α*1 gene expression induced by TGF-*β*1. In rat mesangial cells cultured in diabetic conditions or pretreated with TGF-*β*1, expressions of a methyltransferase SET7 increased, along with the enrichment of SET7 at fibrotic gene Col1a1/Col4a1 promoters. Contrarily, SET7 gene silencing suppressed Col1a1/Col4a1 gene expression [72].

In addition, in high-glucose-treated mesangial cells and diabetic animals, histone modifications stimulated the transcription of fibronectin gene and ECM accumulation, ultimately promoted the progression of renal fibrosis. This is supported by the observation that in rat mesangial cells pretreated TGF-*β*1 and high glucose, the H3K9/14Ac and HAT p300/CBP assembled at promoters of fibronectin-1 (FN-1) gene, which will bring about the facilitation of FN-1 expression [62]. In another study, it was noted that renal fibronectin-1 (FN-1) gene expression in type 1 diabetic mice was augmented by the enrichment of H3K9/14Ac and HAT p300/CBP at the promoters of the FN-1 gene, which would lead to the development of renal fibrosis and expression of DKD [64].

Both cell culture and animal studies lent support to the effect for HDACs in the regulation of ECM collection and renal fibrosis in DKD [73]. Moreover, a recent study revealed the expression of various HDACs in kidneys of patients with diabetes and streptozotocin- (STZ-) induced diabetic rats, which prove that HDAC4 plays a considerable role in the progression of DKD [74].

## 9. Histone Modifications Stimulate EMT Progress

EMT is widely recognized as an important mechanism that could result in the transformation of injured renal tubular cells into mesenchymal cells. This transition from renal tubular cells to mesenchymal cells may result in the renal dysfunction all throughout the nephron in chronic renal failure including DKD [18, 31]. Histone modification is similarly involved in kidney fibrosis through the progression of EMT. Yoshikawa et al. [75] reported the global reduction of heterochromatin marker H3K9Me2, increase of euchromatin marker H3K4Me3, and increase of the transcriptional marker H3K36Me3 during the EMT progress, which are mostly dependent upon lysine-specific deaminase-1 (Lsd1). Studies in a unilateral urethral obstruction model revealed that TGF-*β*1-induced EMT in rat tubular epithelial cells could be blocked by trichostatin A (TSA), a nonselective HDAC inhibitor, leading to the suppression of fibronectin and *α*-SMA gene expression in the kidney [76]. Additionally, TSA inhibited TGF-*β*1-stimulated EMT in human proximal convoluted epithelial cells [75]. Similar results have also been reported in STZ-induced diabetes and TGF-*β*1-intervened tubular epithelial cells from normal rat kidney (NRK52-E), indicating that HDAC-2 has an important role in the progression of DKD [77].

## 10. TGF-***β*** Signaling Pathway Regulates the Histone Modifications

TGF-*β* signaling is considerable in the stimulation of the expression of fibrotic and ECM genes associated with changes in posttranscriptional histone modifications in diabetes or hyperglycemia conditions. TGF-*β*1 regulates fibrotic gene expression by activating transcription factors including Smad2, Smad3, and Smad4, collaborating with HATs and chromatin remodeling factors. This viewpoint is supported by the observation that TGF-*β*1 and high-glucose treatment led to the enrichment of H3K9/14Ac and HAT p300/CBP interaction with Sp1 and Smad binding sites at promoters of PAI-1 and p21 gene in rat mesangial cells, along with the enhancement of the interaction between p300 and Smad2/3 and Sp1, as well as the increase of Smad2/3 acetylation, followed by the facilitation of PAI-1 and p21 production [62]. In a rat mesangial cell culture model, the increased expression of Col1a1, PAI-1, and CTGF genes induced by TGF-*β*1 was associated with elevated levels of active Kme marks (H3K4me1, 2, and 3) and reduced levels of inhibitive marks (H3K9me2 and 3) at their promoters and was accompanied with the accumulation of HMT SET7/9 to fibrotic and ECM gene promoters [63]. Conversely, increased fibrotic and ECM levels induced by hyperglycemia and changes in promoter H3Kac and H3Kme, as well as SET7 recruitment, were significantly blocked by TGF-*β*1 antibody treatment, emphasizing the significant role of TGF-*β*1 in hyperglycemia-induced epigenetic histone modifications [62, 63]. A significant increase in HDAC-2 activity has been reported in kidneys of diabetic rats induced by STZ and db/db mice, as well as TGF-*β*1-treated NRK52-E cells [77]. In addition, MS-275, a selective inhibitor of class I HDAC, reversed to a significant degree of fibrosis in DKD by inhibiting TGF-*β* signaling and renal fibroblast activation [78]. Thus, TGF-*β*1-induced EMT progress and histone modification seem to have a significant role in the accumulation of ECM and tubular interstitial fibrosis [75–77, 79]. These indicate that histone modification modulates renal fibrotic and ECM gene expression under diabetic conditions through TGF-*β* signaling (Figure 2).

## 11. Epigenetic Therapies in Diabetic Kidney Disease to Suppress Renal Fibrosis

In view of the evidences discussed in the preceding section, attempts are being made to inhibit the activities of HAT, HDAC, and HMT, in order to suppress DKD (Table 1). Curcumin, a HAT p300 inhibitor, prevented high-glucose-induced changes in gene transcription levels associated with the downregulation of histone acetylation [80, 81], although further studies revealed that curcumin failed to attenuate albuminuria associated with diabetes mellitus [82]. In contrast, curcumin analog C66 significantly and persistently prevented renal fibrotic gene expression in diabetic mice by inhibiting diabetes-associated increases in p300/CBP expression, HAT activities, and histone acetylation [64]. Furthermore, emerging evidences have shown that HDAC inhibitors with protective effects on kidneys could serve as potential antifibrotic molecules in DKD (Table 1). However, it remains unclear whether the effects of HDACs are due to the inhibition of epigenetics or nonepigenetics [73]. Valproic acid (VPA), an antiepileptic and antimigraine drug, is a nonspecific HDAC inhibitor. A recent study revealed that VPA treatment significantly suppressed histological alterations and fibrosis in diabetic rat kidneys and decreased the fibrotic gene expression and accumulation of ECM proteins [83]. In kidneys of STZ-induced diabetic rats, TSA suppressed the mRNA and protein expression of the constituents of the ECM and ameliorated the EMT progress [77]. Similar beneficial actions were observed in NRK52-E cells in vitro with VPA and class I HDAC-selective inhibitor SK-7041. Due to the complexity of histone methylations and the multiplicity of HMTs, the effects of HMT inhibitors in the renal fibrosis of DKD remain unclear. However, a recent study revealed that histone demethylase JMJDA2A inhibition achieved by chemical inhibitor 2,4-PDCA and siRNA suppressed VSMC migration, proliferation, and inflammation caused by hyperglycemia in vitro and mitigated neointimal formation in balloon-injured diabetic rats [84].

## 12. Conclusion

The pathogenesis of DKD is complex, in which interactions among injury factors, growth factors/cytokines, ROS, inflammation and fibrosis participate in several signal transduction pathways. Chronic, relentless renal fibrosis and ECM accumulation are pathologic features of DKD. At present, it is widely believed that the TGF-*β* pathway, which triggers EMT progress, plays a significant role in the accumulation of ECM proteins in DKD. A number of investigations have confirmed the presence of “metabolic memory” in the progression of diabetic complications including DKD. Over the past years, epigenetic factors have been implicated in “metabolic memory” and diabetic complications, as described above. Accumulating evidence suggests that in diabetes and hyperglycemia conditions, epigenetic histone modifications play a considerable role in modulating kidney gene expression, which contributes to renal fibrosis and ECM accumulation. Histone modification is likewise involved in kidney fibrosis through the regulation of EMT progress. In addition, TGF-*β* signaling can stimulate the expression of fibrotic and ECM genes correlated with changes in posttranscriptional histone modifications induced by diabetes or hyperglycemia. As histone modifications are implicated in the progression of kidney fibrosis, several HDAC, HAT, and HMT inhibitors are currently being advanced for the management of DKD, which could attenuate fibrogenesis. Hence, it is imperative to understand the consequences of variations in histone lysine acetylation and methylation, in order to explore novel biomarkers and therapeutic directions for DKD.

## Figures and Tables

**Table 1:** Effects of HDAC inhibitors on diabetic kidney disease.

HDAC inhibitors	Selectivity	Experimental model	Effects	Mechanism	References
Valproic acid	HDAC I/II	STZ-induced diabetic rat kidneys and TGF-*β*1-treated NRK52-E cells	Decreases ECM components and prevents EMT	Suppresses TGF-*β*1-induced activation of HDAC-2	[77]
		STZ-induced diabetic kidneys	Alleviates the renal damage and fibrosis	Repressing the myofibroblast transformation and fibrogenesis	[83]
TSA	HDAC I/II	STZ-induced diabetic rat kidneys and TGF-*β*1-treated NRK52-E cells	Decreases ECM components and prevents EMT	Suppresses TGF-*β*1-induced activation of HDAC-2	[77]
		TGF-*β*1-treated RMCs	Further increased TGF-*β*1-stimulated PAI-1 gene transcriptional capability and expression	Further amplified TGF-*β*1-motivated H3K9/14Ac levels	[62]
SK7041	HDAC I	STZ-induced diabetic rat kidneys and TGF-*β*1-treated NRK52-E cells	Decreases ECM components and prevents EMT	Suppresses TGF-*β*1-induced activation of HDAC-2	[77]
Vorinostat	HDAC I/II	Cultured proximal tubule cells and STZ-induced diabetic kidneys	Attenuated cellular proliferation, suppressed glomerular hypertrophy	Downregulated EGFR expression	[85]
		STZ-diabetic mice	Decreased oxidative stress, albuminuria, and collagen IV deposition	Interplay between eNOS activity and oxidative stress	[86]
Sodium butyrate (NaB)	Pan HDAC inhibitor	STZ-induced diabetic kidneys	Ameliorated renal function and relieved histological alterations, apoptosis, fibrosis, and DNA damage	NA	[87]

## Abbreviations

*α*-SMA: *α*-Smooth muscle actin
BMP-7: Bone morphogenic protein 7
CTGF: Connective tissue growth factor
DKD: Diabetic kidney disease
ECM: Excess extracellular matrix
EMT: Epithelial-to-mesenchymal transition
FN-1: Fibronectin-1
HAT: Histone acetyltransferase
HDAC: Histone deacetylase
HMT: Histone methyltransferase
MRTF-A: Myocardin-related transcription factor A
PAI-1: Plasminogen activator inhibitor 1
Smad: Mothers against decapentaplegic homologue
STZ: Streptozotocin
TGF-*β*1: Transforming growth factor *β*1
TSA: Trichostatin A.
